# Family Issues in Japanese Clinics: Concordance between Patients’ and Physicians’ Views

**DOI:** 10.31662/jmaj.2019-0074

**Published:** 2021-07-09

**Authors:** Hiroaki Takenaka, Nobutaro Ban, Tomoyuki Kido, Shinji Takeda, Juichi Sato

**Affiliations:** 1 Takenaka Clinic, Osaka, Japan; 2 Medical Education Center, Aichi Medical University School of Medicine, Nagakute, Japan; 3 Aikoen Clinic, Osaka, Japan; 4 Higashimachi Family Clinic, Iwamizawa, Japan; 5 Department of General Medicine/Family and Community Medicine, Nagoya University Graduate School of Medicine, Showa, Japan

**Keywords:** Family Health, Physician-Patient Relations, Family Practice, Behavioral Medicine, Behavioral Research, Outpatients

## Abstract

**Introduction:**

The objectives of the present study were to clarify the frequency and content of family issues for patients in Japanese clinics, and the concordance between physicians’ and patients’ views of family issues.

**Methods:**

In this study, we used a cross-sectional design with a questionnaire survey. Participants were outpatients and their physicians in charge (four family physicians) at four Japanese clinics. The main body of research was conducted between April 5 and May 15, 2004. After obtaining oral informed consent, the physician in charge distributed questionnaires to participating patients to complete at home. The questionnaire comprised three items: 1) Do you have any worries about your family? 2) Are you comfortable consulting a physician regarding your family issue?, and 3) If possible, could you tell us why you feel like that?

Participants provided written informed consent and answered the questionnaire before sealing it in an envelope and posting it back to the research center. Physicians in charge completed their version of the questionnaire and independently sent the data to the center.

**Results:**

Of the 272 participating patients, 118 (45.6%) had family issues. “Health problems with family members” (28%) and “family life cycle issues” (19.5%) were the main content of these issues. Physicians indicated that 45.7% of patients had family issues. The rate of concordance between physicians’ and patients’ perspectives regarding family issues was 46.6%.

**Conclusions:**

Family issues can therefore be regarded as a common health problem due to the frequency. There was some inconsistency between physicians’ and patients’ views, but much of this discrepancy may be resolved by developing the specialty of family practice.

## Introduction

In Japan, family physicians deal with common health problems. Some Japanese family physicians indicated that they were not interested in a family approach because they viewed family issues as a specialist area beyond the scope of family medicine ^(1), (2)^. Furthermore, as there is no clear definition of “family” or “family issues,” it is difficult to produce research results and obtain study funding in this scientific field. This means that little research on family issues has been conducted, and the research field is underdeveloped. In this context, family physicians may believe that they do not need to consider family issues, especially if they are uncommon. We found five previous studies that investigated whether family issue was common in relation to health problems. In the US, two studies were conducted in clinics and involved retrospective chart reviews. One investigation by Merenstein et al. showed that family issues were recorded in 12% of the charts ^(3)^. The second investigation by Beasley et al. found that family issues were recorded in 8% of the charts in facilities associated with the division of family practice ^(4)^. Three previous Japanese cross-sectional studies were conducted in a university ^(5)^ and two community hospitals ^(6), (7)^. Those studies reported that approximately 30% of patients had family issues regardless of place or size of the hospital. These studies raised the question as to why the frequency of family issues differs so markedly between the two countries. A conceivable reason is the different settings, and the US studies were retrospective chart surveys conducted in clinics (physicians’ views). By contrast, the Japanese studies were conducted in hospitals and used cross-sectional designs (patients’ views).

The first objective of the present study is to clarify the frequency and content of family issues in Japanese clinics from the patients’ perspective. The second objective is to consider family issues as determined from the physicians’ perspective. The third objective is to measure the rate of concordance regarding family issues between physicians and patients. The last objective is to clarify the kind of family issues that patients found difficult to consult with their physicians.

## Materials and Methods

This study used a cross-sectional questionnaire survey design. Participants were outpatients and their physicians in charge (four family physicians) at four different Japanese clinics. A pilot study was conducted on January 19-24, 2004, with the main body of research conducted between April 5 and May 15, 2004.

### Participants

The study population was outpatients from Japanese clinics. The sample comprised outpatients from four clinics that had a family physician who was familiar with research. We chose clinics from four different areas of Japan to avoid bias relating to regional characteristics.

The eligibility criterion was randomly selected outpatients by the research center. The exclusion criteria were patients who did not want to participate, could not understand Japanese characters, could not understand the study content, had communication difficulties, and for whom participation was judged as unfavorable therapeutically by the physician in charge.

### Methods

After obtaining oral informed consent from patients, the physician in charge distributed the questionnaire to patients to complete at home. Participating patients then provided written informed consent and answered the questionnaire, after which they sealed it in an envelope and posted it back to the research center. The questionnaire comprised three items: 1) Do you have any worries about your family? 2) Are you comfortable to consult a physician regarding your family issue?, and 3) If possible, could you tell us why you feel like that? (Refer to Supplement).

In addition, the physicians in charge independently completed the physicians’ version of the questionnaire and sent the data to the center. The physicians’ questionnaire covered the patients’ primary diseases, the family issues that they assessed, and the intervention methods. Participating physicians reviewed their patients’ charts to answer the questionnaire. The physicians were also asked to calculate the rate of description if they did not describe all of the contents of family issues that they perceived were experienced by their patients.

### Handling of missing data

Missing data were defined as data for patients who met the exclusion criteria and did not return a questionnaire. These data were excluded from the analysis.

### Ethical considerations

Written informed consent was obtained from all participants. The Institutional Review Board of Nagoya University approved the study protocol (No. 148003). The authors declare that they have no conflicts of interest.

## Results

Participants’ demographic data are shown in Table 1. Of the 429 eligible patients, 341 provided informed consent and were given a questionnaire. In total, 272 replies were received by the research center (recovery rate, 79.8% (272/429)) from 117 men and 155 women. Participants’ mean age was 64.3 years (standard deviation [SD], 14.4 years).

**Table 1.** Characteristics of This Study.

• Subjects	429
• Registrants	341
• Participants	272
* Valid response rate (Participants/Subjects) = 63.4%	
Of the participants, Gender	
Gender	
Male	117
Female	155
Mean age	64.3 years (SD 14.4)
Average number of family members	3.0 (SD 1.5)

The average number of family members per participant was 3.0 (SD, 1.5). Participants’ family life cycle stages were the following: couples (n = 2), newly married (n = 4), perinatal family (n = 4), families with small children (n = 9), families with school-aged children (n = 9), families with young adolescents (n = 11), middle-aged families without offspring (n = 1), families with older children that had left home (n = 34), aged family (n = 113), widowhood (n = 60), unmarried (n = 3), and nonresponders (n = 22) (Table 2).

**Table 2.** Family Life Cycle Stages of Participants and Contents of Family Life Cycle Issues.

Family lifecycle stages (n = 272)		Family lifecycle issues (n = 23)	
• Couples	2	• Marriage issues	7
• Newly married	4	• Childbirth issue	1
• Perinatal family	4	• Childcare issues	5
• Families with small children	9	• Issues with advancing to higher learning	3
• Families with school-aged children	9	• Issues with adolescence	2
• Families with young adolescents	11	• Issues with retirement or career change	2
• Middle-aged families without offspring	1	• Aging issue	1
• Families with older children that had left home	34	• Caregiving issue	2
• Aged family	113		
• Widowhood	60		
• Unmarried	3		
• Non-responder	22		

**Figure 1.**
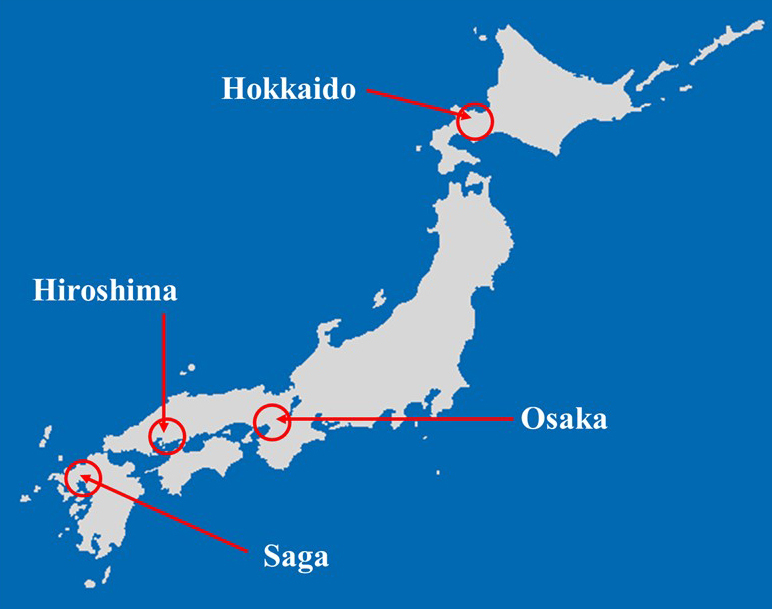
Location of the four participating clinics.

Participating physicians were four Japanese family physicians. All physicians were men, and their mean age was 46.7 years (SD, 2.5 years). They were top-level family physicians because there were few family physicians that were familiar with the survey at that time in Japan. The four participating clinics were located in Hokkaido, Osaka, Hiroshima, and Saga (Figure 1). These clinics were selected to address the issue of regional differences.

### Frequency and content of family issues

**Figure 2.**
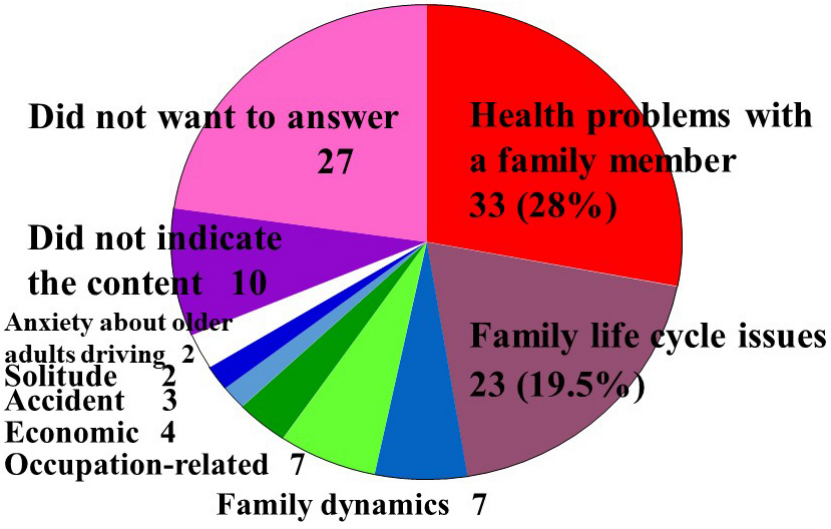
Contents of family issues.

We found that 118 participants reported family issues (45.6% (118/272)), which was a higher rate than that reported in previous studies in Japanese hospitals or US clinics (Table 3). Around half (51.8% (141/272)) of the participating patients had no family issues, and 13 (2.6%) did not reply. The most common family issue content was “Health problems with family members” (28% (33/118)). “Family life cycle issues” was the second most common type of issue (19.5% (23/118)). Family life cycle refers to a series of stages through which a family may pass over time. In each stage, a family faces challenges that allow them to build or gain new skills and issues may appear if family members are not able to build or gain these new skills. In this study, we identified 23 family life cycle issues (Table 2), including the following: marriage issues (n = 7), childbirth issues (n = 1), childcare issues (n = 5), issues with advancing to higher learning (n = 3), issues with adolescents (n = 2), issues with retirement or career change (n = 2), aging issues (n = 1), and caregiving issues (n = 2). These two issues (Health problems with family members and Family life cycle issues) accounted for nearly half of all issues. Other issues included the following: issues in family dynamics (n = 7), including discord between family members and divorce/separation of a family member; occupation-related issues (n = 7) such as layoff from a job; economic issues (n = 4), including hospitalization costs, mortgage, inheritance issues, and waste of money by a family member; solitude (n = 2); accidents (n = 3), including accidental death, disappearance of a family member, and imprisonment of a family member; and anxiety about older adults driving (n = 2). Ten patients did not indicate the content of their issue, and 27 did not want to answer (Figure 2).

**Table 3.** Comparison between This Research and Previous Ones.

Physicians’ view		
This research (Japan)	45.7%	(n = 92)
Merenstein, et al (US)	11.9%	(n = 1420)
Beasley, et al (US)	8.0%	(n = 1126)
Patients’ view	
This research (Clinic)	45.6%	(n = 272)
University Hosp.	25.4%	(n = 250)
Community Hosp.
*Diabetic outpatient*	35.5%	(n = 120)
*Surgical outpatient*	23.7%	(n = 135)

### Physicians’ perspective

Information obtained on family issues from the physicians’ perspective concerned 92 patients (recovery rate, 27.0% (92/341)). The four participating physicians judged that 42 of these patients (45.7% (42/92)) had family issues. This rate was as high as that noted by patients themselves. Two of the physicians calculated the rate of description of family issues on their charts, which indicated that 50%-80% of family issues were recorded. The other two physicians did not calculate the rate of description. This revealed the following 32 family issues (Table 4): health problems with family members (n = 15); family life cycle issues (n = 10), including six deaths of family members (three husbands, one wife, one mother, and one son), two aging issues, one sharing childcare issue, and one issue regarding a successor for farms; issues in family dynamics (n = 5), including discord between couples (n = 2), parent-child discord (n = 1), divorce of a family member (n = 1), and sharing housework (n = 1); employment of a son (n = 1); and solitude/no caregiver (n = 1).

**Table 4.** Contents of Family Issues from the Perspective of Doctors in Charge.

	(n = 32)		
• Health problems with family member	15	• Family life cycle issues	10
Care for the partner	3	Death of family members	6
COPD, Depression, Unknown reason		(husband 3, wife, mother, son)	
Care for the parent (Three mother and one father)	4	Aging issues	2
Cerebral infarction, Unknown reason		Sharing child care issues	1
Care for the son	2	Issues regarding a successor for farms	1
Depression, Unknown reason		• Issues in family dynamics	5
Care for the grandchild	1	Discord between couples	2
Congenital abnormality		Parent-child discord	1
Terminal care	1	Divorce of a family member	1
Prevention of nosohusial infection of common cold	1	Sharing housework	1
Non-adherence (Hospitalization, Alcohol)	2	• Employment of a son	1
Allergic constitution	1	• Solitude/No care-giver	1

Specific issues relating to health problems with family members included the following: care for a partner (n = 3) because of chronic obstructive pulmonary disease (COPD), depression, and an unknown reason; care for parents (n = 4), including three mothers and one father (one cerebral infarction and three unknown); care for a son (n = 2) because of depression and an unknown reason; care for a grandchild with congenital abnormality (n = 1); terminal care (n = 1); prevention of nosohusial infection of common cold (n = 1); nonadherence (n = 2), including hospitalization and alcohol-related issues; and allergic constitution (n = 1). There were no details provided for the other 11 types of family issues.

### Concordance between patients’ and physicians’ views

There were 73 valid responses. In 11 cases, both patients and physicians listed the same issue, and in 23 cases, both parties listed no issue. This meant that physicians and patients agreed in 34 cases, giving a concordance rate of 46.6% (34/73). The issues on which patients and physicians agreed were the following: health problems with family members (n = 8); family life cycle issues (n = 4), including two deaths of family members (one husband and one son), one aging issue, and one sharing childcare issue; issues in family dynamics (n = 2), including parent-child discord and sharing housework; employment of a son (n = 1); and solitude/no caregiver (n = 1). Health problems with family members included the following: care for a partner (n = 3) because of COPD, depression, and an unknown reason; care for a parent (n = 3), including two mothers and one father (cerebral infarction and two unknown); care for a son (n = 1) with depression; and care for a grandchild (n = 1) with congenital abnormality.

One of the inconsistency between physicians’ and patients’ reports concerned cases in which the patient acknowledged family issues but the physician did not. These issues included the following: health problems of family members (n = 5); issues in family dynamics (n = 4), including discord between family members and divorce of a family member; family life cycle issues (n = 3); occupation-related issues (n = 2); and waste of money by a family member (n = 1). Other cases of disagreement involved the physician acknowledging family issues when the patient did not. These issues included the following: health problems of a family member (n = 4) comprising poor adherence relating to alcohol and refusal of hospitalization, caregiving fatigue, and allergic constitution; family life cycle issues (n = 3), including deaths of family members and the lack of a successor for farms; and issues in family dynamics (n = 2) comprising discord between family members and divorce of a family member (Table 5).

**Table 5.** Inconsistency.

	(n = 31)		
• Patients acknowledged family issues, but Physicians didn’t (18 cases)		• Physicians acknowledged family issues but patients didn’t (13 cases)	
Health problems of family members	5	Health problems of a family member	4
Issue in Family dynamics	4	Poor adherence	2
Discord	3	relating to alcohol	1
Divorce	1	Refusal of hospitalization	1
Family lifecycle issues	3	Caregiving fatigue	1
Occupation-related issues	2	Allergic constitution	1
Waste of money by a family member	1	Family lifecycle issues	3
Unable to answer	3	Death of family members	2
No answer	1	Lack of a successor for farms	1
		Issue in Family dynamics	2
		Discord	1
		Divorce	1
		No answer	4

### Family issues difficult for patients to consult about

There were 76 valid responses concerning issues that participants found difficult to consult with their doctor, including the following: being in consultation (n = 34), attempting to consult (n = 8), unable to consult (n = 27), and not sure (n = 7). This meant that 60.9% (42/69) of patients reported that family issues were easy to consult with a physician and 39.1% (27/69) found it difficult to consult with their physician about these issues. For patients, family issues that were easy to consult with their family physicians about were the following: health problems with a family member (n = 18), family life cycle issues (n = 5), divorce of a family member (n = 2), discord between family members (n = 1), disappearance of a family member (n = 1), and imprisonment of a family member (n = 1). Family issues that patients found difficult to consult with their family physicians were the following: family life cycle issues (n = 6), health problems with a family member (n = 4), discord between family members (n = 1), divorce of a family member (n = 1), waste of money by a family member (n = 1), mortgage (n = 1), occupation-related issues (n = 1), and anxiety about an older adult driving (n = 1).

There were 22 valid responses for reasons why patients found it difficult to consult with their physician. These were the following: no relationship to the disease (n = 6), unable to be resolved by a family physician (n = 4), too private (n = 3), not serious enough to consult (n = 3), presence of another physician in charge of the family member (n = 2), problem that should be solved by oneself (n = 2), not certain whether it was suitable to consult a physician (n = 1), and no particular reason (n = 1). Underlying resources that became clear were noted in seven cases. For example, “no relationship to the disease” concerned a relative (e.g., marriage of a daughter) and a friend (e.g., adolescent problems), “too private” concerned friends (e.g., layoff from a job and issues with advancing to higher learning), “not serious enough to consult” concerned a friend (unable to answer), “problem that should be solved by oneself” concerned a brother, and “not certain whether it was suitable to consult a physician” concerned a mother (i.e., care of the father).

## Discussion

Our data suggest that 45.6% of outpatients in Japanese clinics had some family issues, and such issues can therefore be regarded as a common health problem. The contents of family issues in Japanese clinics were similar to those reported in hospitals in previous Japanese studies. However, the incidence of family issues in this study involving Japanese clinics was higher than that reported in the US and in Japanese hospitals (Table 3). We found a relatively high concordance rate between physicians’ and patients’ views (46.6%) regarding family issues. In total, 57.6% (38/66) of patients reported that family issues were easy to consult with a physician, and 42.4% (28/66) found it difficult to consult with their physician about these issues. The main reasons why patients found it difficult to consult with their physician were “no relationship to the disease,” “unable to be resolved by a family physician,” “too private,” “not serious enough to consult,” “presence of another physician in charge for the family member,” “problem that should be solved by oneself,” “not certain whether it was suitable to consult a physician,” and “no particular reason.”

We considered three possible reasons why the frequency of family issues in clinic settings differed so markedly between Japan and the US. The first possible reason may be the different culture surrounding family in the two countries. Health problems with family members were mainly family issues reported by participants in this study. There is a strong belief in Japan that the family should take responsibility for the care of children and elderly family members. Of course, there is also a considerable care burden in the US, but the family structure and culture in Japan tends to make the care burden stronger. Therefore, it may be that health problems with family members in Japan may be more easily actualized. In addition, good access to medical care may make it easy for doctors to recognize such problems in Japan, especially with a national health insurance system. The second possible reason is the different study designs. Obviously, retrospective chart reviews will identify fewer family issues. In this study, two investigators (family physicians) reviewed the charts and calculated the rate of description. As a result, 50%-80% of family issues were recorded. Possibly, differences in medical education between Japan and the US in terms of charting mention of family issues might have influenced the results. However, we could not find previous research on this topic and did not have sufficient data in this study. Further studies are needed to clarify this point. The third possible reason is different definitions of family issues between the two countries. To our knowledge, there is no consensus about an appropriate definition of “family issue.” Currently, three different types of definition can be considered. The first is a subjective concept of family issues; when a patient says, “we have family issues,” we take their words for granted. Another definition is from the perspective of medical staff; when medical staff feels that “this family has family issues,” the patient is categorized as having family issues. Finally, family issues can be defined as someone who may be a patient, their family member, a medical staff, or a third party and who feels “this family has family issues.” Furthermore, there is no clear definition of family and family issues, which means that obtaining research funding and producing results in this field are difficult. As a result, there is little research in this underdeveloped field.

With regard to the higher incidence of family issues in Japanese clinics in this study (45.6%) than that reported in previous hospital-based studies (Table 3), a conceivable reason may be the present investigators’ (leaders in Japanese family medicine) level of interest in family issues. However, the exact reason has not been confirmed.

In this study, “issues in family dynamics” was a common family issue for which physicians’ and patients’ views differed. This type of issue might be difficult for Japanese family physicians to identify correctly. “Family death” was another point of inconsistency, although this type of issue might have been more likely to be detected by physicians when considering the necessity of grief work. In addition, issues such as poor adherence and allergic constitution were recognized by physicians but not by patients.

Many patients in this study reported experiencing difficulty consulting with their physician about some issues, although some issues reported to be difficult (e.g., family life cycle issues and anxiety about older adults driving) are within the primary scope of family physicians’ care. However, patients did not wish to consult with family physicians about these issues because they felt that the issue had no relationship to their disease or were uncertain whether it was suitable to consult about such issues with their physician. With regard to issues such as “discord between family members,” “divorce of a family member,” “waste of money by a family member,” and “mortgage,” family physicians can help patients with these issues through collaboration with other specialists, such as family therapists and social workers. Many issues that patients felt were inappropriate to consult with a physician were within the primary scope of the family practice specialty, which suggests that we should further develop the specialty of family practice in Japan. Strengthening the specialty may help in resolving the majority of the inconsistencies identified in this study.

Lastly, our study had several limitations that should be considered. A major limitation concerns the publication of our results, which took a considerable amount of time because two coinvestigators unfortunately passed way during the analysis period of the study. Considering the extensive gap between data collection and publication, it is possible that the Japanese family structure and social context differ today compared with when the study was first conducted. In addition, all authors who participated in this study were practicing physicians in small-scale clinics where only one or two full-time physicians were engaged in daily clinical practice, which made it difficult to complete the manuscript. However, our study remains important despite the time taken to publish it, and we are releasing these results because of their importance.

As of 2021, it is thought that the number of doctors adopting a family approach has increased. Doherty WJ, Baird MA (1986) ^(8)^ and Takenaka H, and others (2016) ^(9)^ identified five different levels of physician involvement with patients and their families. The approach of Level 1 and 2 has been performed widely. However, the use of higher-level techniques has not shown the same progress. The selected clinics had Japanese family physicians that had top-level clinical techniques and were familiar with this study. Their technique appeared to be almost the same as the level in 2021. However, this might reflect selection bias and affect the generalization of our results.

In addition, limitation of this study was that selection bias and response bias might have influenced the results by excluding missing data. Although we addressed the issue of regional differences, there was a little deviation to West Japan.

Finally, our sample was small, the ratio of men to women was 117:155, and participants only included Japanese outpatients. We hope subsequent studies will be conducted in cooperation with researchers in other countries to overcome these limitations.

## Conclusions

Family issues are a common health problem reported by almost half (45.6%) of outpatients visiting Japanese clinics. Contents of “family issues” in Japanese clinics are similar to those previously reported in Japanese hospitals. The concordance rate between physicians’ and patients’ views is relatively high (46.6%), but patients regard around 8.7% of family issues as difficult to consult with family physicians. However, these issues are mostly within the primary scope of family practice. Therefore, the majority of these issues may be resolved by further developing our specialty.

## Article Information

### Conflicts of Interest

None

### Acknowledgement

We appreciate the work of everyone who supported this study, especially Dr Yoshikazu Tasaka and Dr Masashi Shirahama who were investigators in this study but unfortunately passed away in the middle of developing this article. Our condolences go to both families.

### Author Contributions

Dr Takenaka is the corresponding author. He made substantial contributions to the analysis and interpretation of the data and wrote this manuscript.

Dr Ban was involved in drafting the manuscript and revising it critically for important intellectual content and provided final approval of the version to be published. 

Dr Kido and Dr Takeda contributed to collecting written informed consent and data acquisition.

Dr Sato made substantial contributions to the conception and design of this study.

### Approval by Institutional Review Board (IRB)

The Institutional Review Board of Nagoya University approved the study protocol (No. 148003).

### Prior Presentations

Frequency and contents of family issues: 17th WONCA World Conference of Family Doctors. Oct 14, 2004, Orlando, US.

Concordance of family issues between physicians’ and patients’ views: 14th WONCA Asia Pacific Regional Conference. May 28, 2005, Kyoto, Japan.

## Supplement

Supplementary Material
