# Supplementary material for: Family Issues in Japanese Clinics: Concordance between Patients’ and Physicians’ Views
Source: JMA J. 2021 Jul 9;4(3):254–61. doi: 10.31662/jmaj.2019-0074 (PMC8355727; doi:10.31662/jmaj.2019-0074)
Supplement: Supplementary file 1 — Supplementary Material [file 2433-3298-4-3-0254-s001.pdf]

Supplement The questionnaire

(We modified the original version written in Japanese into English)

Please answer the following questions

Question 1: Do you have any worries about your family? Please circle the applicable section.

（　　　　　） No　　　　→Please proceed to the third page

（　　　　　） I have some worries, but I don't want to describe it.

→Please proceed to the Question 2

（　　　　　） Yes　　　　→Please fill in below to describe the content and proceed to the Question 2

Question 2: (Only for those who answered "yes" or "I have some worries, but I don't want to describe it" to the Question 1)

Are you comfortable to consult a physician regarding your family issue? Please circle the applicable section.

- (   ) It is currently being consulted with the doctor in charge.
- (   ) I will probably consult with the doctor in charge about it.
- (   ) I'm not sure about that.
- (   ) I am not able to consult with the doctor in charge about it.

→Who is the person whom you can consult?

If possible, could you tell us why you feel like that?

Finally, please tell us your age and family structure.

You are ______________ years old Male ・ Female

There are _________ people in my family.

Please fill in below to describe your family structure

We would appreciate it very much if you could point out anything to us.

You put this paper in the envelope which we handed, and please mail it after sealing it.

We appreciate your cooperation very much.
